# Hydrogen Permeation Behavior of Zirconium Nitride Film on Zirconium Hydride

**DOI:** 10.3390/ma15020550

**Published:** 2022-01-12

**Authors:** Wenke Wang, Guoqing Yan, Jiandong Zhang, Zhaohui Ma, Lijun Wang, Zhancheng Guo, Shunli Zhang, Yanke Wu

**Affiliations:** 1 National Engineering Research Center for Environment-Friendly Metallurgy in Producing Premium Non-Ferrous Metals, GRINM Group Corp. Ltd., Beijing 101407, China; wwk18801271070@163.com (W.W.); 18500936007@163.com (G.Y.); zjd332@163.com (J.Z.); mazhaohui@grinm.com (Z.M.); 18611123468@163.com (S.Z.); luckwyk@163.com (Y.W.); 2 State Key Laboratory of Advanced Metallurgy, University of Science and Technology Beijing, Beijing 100083, China; zcguo@ustb.edu.cn; 3 GRINM Resources and Environment Tech. Co. Ltd., Beijing 101407, China; 4 General Research Institute for Nonferrous Metal, Beijing 100088, China

**Keywords:** zirconium hydride, zirconium nitride film, hydrogen permeation mechanism, hydrogen permeation rate, in-situ reaction, neutron moderator

## Abstract

Hydrogen permeation barrier plays an important role in reducing hydrogen loss from zirconium hydride matrix when used as neutron moderator. Here, a composite nitride film was prepared on zirconium hydride by in situ reaction method in nitrogen atmosphere. The phase structure, morphology, element distribution, and valence states of the composite film were investigated by XRD, SEM, AES, and XPS analysis. It was found that the composite nitride film was continuous and dense with about 1.6 μm thickness; the major phase of the film was ZrN, with coexistence of ZrO_2_, ZrO, and ZrN_0.36_H_0.8_; and Zr-C, Zr-O, Zr-N, O-H, and N-H bonds were detected in the film. The existence of ZrN_0.36_H_0.8_ phase and the bonds of O-H and N-H revealed that the nitrogen and oxygen in the film could capture hydrogen from the zirconium hydride matrix. The hydrogen permeation performance of nitride film was compared with oxide film by permeation reduction factor (PRF), vacuum thermal dehydrogenation (VTD), and hydrogen permeation rate (HPR) methods, and the results showed that the hydrogen permeation barrier effects of nitride film were better than that of oxide film. The zirconium nitride film would be a potential candidate for hydrogen permeation barrier on the surface of zirconium hydride.

## 1. Introduction

The type of reactor included gas cooled reactor (GCR), light water reactor (LWGR), heavy water reactors (PWR), and fast reactors (FRs) [1]. The space reactor included thermal neutron reactor and fast neutron reactor. There were Romaska, Topaz-I, Topaz-II and Topaz* in Russia’s research on space reactor, and the life of them were from Several months to 7 years. American research on space reactor included SNAP-10A and SNAP-100, and the life of them were 43 days and 7–10 years [2,3].

Because of its high thermal stability, high hydrogen density [4], low neutron capture cross-section, and good thermal conductivity [5,6,7], zirconium hydride became an ideal neutron moderator and was put into application in space nuclear reactor. However, hydrogen will escape from zirconium hydride in the working environment, leading to the loss of hydrogen and hence deteriorating the moderating effect of the moderator [8]. The hydrogen loss can be prohibited or hindered by manufacturing a hydrogen permeation barrier film on the surface of zirconium hydride.

Substantial research has been conducted on the preparation of hydrogen permeation barrier on stainless steel, such as oxide films of Y_2_O_3_, Cr_2_O_3_, and Al_2_O_3_ [9,10,11], carbide films of SiC [12], and nitride films of AlN, Si_3_N_4_, BN, TiAlN [13,14,15,16,17], and PRF values measured were from 40 to 20,000. Zirconium hydride was generally used in honeycomb structure, which made the preparation method of the hydrogen permeation barrier on stainless steel unsuitable for it. Therefore electroplating, micro arc oxidation, sol-gel, and in situ reaction methods were developed to form hydrogen permeation barrier on the zirconium hydride. Chen et al. [18] studied the preparation of the films on zirconium hydride by in situ reaction method in O_2_, CO_2_, and CO_2_ + P atmospheres, and the results showed the M-ZrO_2_ and T-ZrO_2_ film structure, and the films prepared in CO_2_ + P atmosphere had the best hydrogen blocking effect. Wu et al. [19,20] prepared composite oxide films containing Si-Al, Si-Zr, and Si-P on the zirconium hydride by sol-gel method, obtaining a top sol-gel layer and a transition zirconium oxide bottom layer with excellent hydrogen barrier effect. Yan et al. [21] fabricated ZrO_2_ composite film by in situ reaction of urea with zirconium hydride at high temperature, resulting in a film composed of Zr, C, N, and O elements; and C-H, O-H, and N-H bonds were detected in the film, revealing that hydrogen was trapped by C, N, and O in the coating. Qi et al. [22] prepared phosphating film on zirconium hydride, for which the hydrogen permeation rate of samples was measured by gas chromatography, and the hydrogen loss predicted showed a good performance.

Nitride film has also been an important hydrogen barrier category because of its good hydrogen permeation barrier effect, high hardness, and excellent corrosion resistance [23,24]. Methods for preparing nitride film include ion nitriding, carbonitriding and gas nitriding, in which gas nitriding is divided into ammonia nitriding and nitrogen nitriding [24]. Zhang et al. [25] utilized ultrasonic nanocrystalline surface modification (UNSM) technology and low-temperature ammonia nitriding to prepare nitriding layer on the surface of NiCr 718 alloy where the grain boundaries on the surface of the nanocrystals provided the diffusion channels of nitrogen, and this technology reduced the nitriding temperature and time, and improved the hardness, wear resistance, and corrosion resistance of the nitride films. Yilbas et al. [26] prepared nitriding layers on the surface of the tungsten using high-pressure nitrogen with laser assistant, which made the surface of the tungsten nanocrystalline free of microcracks and large-size holes, and the surface hardness was significantly improved, and this nitriding process overcame the binding force of the metal atoms on the metal surface by producing active nitrogen atoms, thus penetrated into the matrix [24]. However, thus far, no research has been published on forming nitride film on zirconium hydride.

In this study, zirconium nitride film was prepared on zirconium hydride by nitrogen nitriding. The phase structure, morphology, element and valence distribution of the film were investigated. The hydrogen permeation performance of nitride film was studied, and was compared with oxide film manufactured by in situ reaction with CO_2_ [18]. The hydrogen permeation mechanism of the nitride film was revealed.

## 2. Materials and Methods

### 2.1. Preparation of Hydrogen Permeation Barrier

Zirconium hydride (H/Zr = 1.8) with the size of Φ 20 mm × 3 mm was prepared by our own research group in National Engineering Research Center for Environment-friendly Metallurgy in Producing Premium Non-ferrous Metals (Beijing, China). Moreover, it was prepared by injecting hydrogen into zirconium niobium alloy, controlling temperature and pressure of the furnace, and zirconium hydride with a certain hydrogen content could be obtained. The purity of zirconium hydride was analyzed by inductive coupled plasma emission spectrometer (ICP), and the contents of O and N in the sample were analyzed by LECO ONH836 analyzer (St. Joseph, MO, USA). The results are shown in Table 1. The elements Al, B, Cd, Co, Cr, Cu, Mg, Mn, Mo, Ni, Pb, Si, Sn, Ti, V and W were all <0.001%. Before the experiment, zirconium hydride was polished to 6.5 μm using a sandpaper step by step, and then cleaned ultrasonically in 99.7% ethanol for 300 s. The power of YA008G ultrasonic cleaning machine (Shenzhen, China) was 80 W. The polished zirconium hydride sample was sealed in quartz tube with 20,000 Pa nitrogen (the purity was 99.99%); subsequently, the packaged quartz tube was placed in a tubular furnace, and the temperature of the furnace was raised from room temperature to 800 °C at 1 °C/min. After keeping for 20 h, the furnace was cooled to room temperature, and the film was obtained.

### 2.2. Characterization of Composition, Morphology and Structure

Scanning electron microscope (SEM, Hitachi S-4800, Tokyo, Japan) was employed to observe the surface and cross-section morphologies of the film. The energy dispersive X-ray spectrometer (EDS) equipped on the SEM was used to characterize the elements distribution of the film. X-ray diffraction (XRD, Smartlab KD2590N, Tokyo, Japan) was employed to investigate the phase of the surface layer. The composition and element distribution of the film was analyzed by Auger electron spectroscopy (AES, ULVAC PHI-700, Chigasaki, Japan). The chemical valence states of the film were analyzed by X-ray photoelectron spectroscopy (XPS, Thermo Scientific™ Nexsa™, Waltham, MA, USA). The hydrogen content of the zirconium hydride before and after in situ reaction was determined by high-temperature dehydrogenation method.

Before the measurement, the samples were all cleaned ultrasonically in ethanol to remove contaminants. Particularly, the samples were measured by SEM to observe cross-section morphology by wire cutting, inlayed with resin, polished to 6.5 μm with sandpaper step by step, and then cleaned ultrasonically in ethanol.

### 2.3. Characterization of Hydrogen Permeation Behavior

To study the hydrogen permeation behavior, the following three methods were employed: (1) permeation reduction factor (PRF) [11]; (2) vacuum thermal dehydrogenation (VTD) [20]; (3) hydrogen permeation rate (HPR) [22]. Hydrogen permeation behavior of nitride film samples prepared in nitrogen atmosphere at 800 °C was studied, and was compared with that of oxide film manufactured by in situ reaction with CO_2_ [18]. The samples investigated are listed in Table 2.

#### 2.3.1. Permeation Reduction Factor

The PRF method was used to evaluate the hydrogen permeation resistance performance by mass loss. The samples with and without film were put into a vacuum furnace and kept at the same set temperature for the same set period of time. The mass loss of samples before and after the experiment was considered to evaluate the hydrogen loss. The definition of PRF was as follows:

(1) $$\mathrm{PRF}=\Delta m_{1}/\Delta m_{2}$$

Δm_1_, mass loss of the sample without film. Δm_2_, mass loss of the sample with film.

In this paper, the samples with and without film were kept in vacuum furnace at 600 °C for 10 h, and the mass of the samples before and after the experiment was measured. The larger the PRF value was, the better the hydrogen permeation resistance was.

#### 2.3.2. Vacuum Thermal Dehydrogenation

The VTD method was employed to assess the hydrogen permeation resistance performance by pressure changes. The zirconium hydride samples with and without film were placed in a tubular furnace and vacuumed to 10^−5^ Pa first, and then the vacuum sample chamber was heated to 600 °C and kept for 10 h, during which the pressure changes with time were recorded. At the same time and temperature, the lower the pressure was, the lower the hydrogen penetration rate was, and the better the performance of the film was in terms of the hydrogen permeation resistance.

#### 2.3.3. Hydrogen Permeation Rate

**Figure 1:**
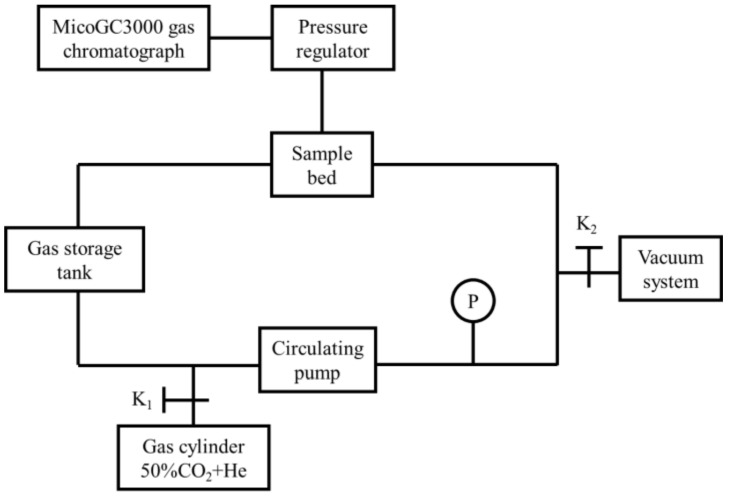
Schematic diagram of hydrogen permeation rate test equipment.

In cooperation with China Institute of Atomic Energy, gas chromatography hydrogen permeability measuring equipment (GCHPME) was used to measure HPR, which consisted of sample bed, gas storage tank, vacuum system, circulating pump, pressure regulator and MicoGC3000 gas chromatograph (Shanghai, China), as shown in Figure 1. Zirconium hydride with different films were placed in GCHPME, and measured at 600 °C with starting atmosphere of He+50%CO_2_ for 7 days. Reactions that may occur during the measurement are as follows:

(2) $${\mathrm{ZrH}}_{x}=\mathrm{Zr}+x/2H_{2}\;\left(g\right)$$

(3) $$H_{2}\left(g\right)+{\mathrm{CO}}_{2}\left(g\right)=\mathrm{CO}\left(g\right)+H_{2}O\left(g\right)$$

During the experiment, the content of H_2_, CO, and CO_2_ in the system were measured by gas chromatography every day, and the content of H_2_ and CO were used to calculate the HPR. Details of the equipment and calculation model are presented by Qi et al. [22] and Bai et al. [27].

## 3. Results and Discussion

### 3.1. Morphology

**Figure 2:**
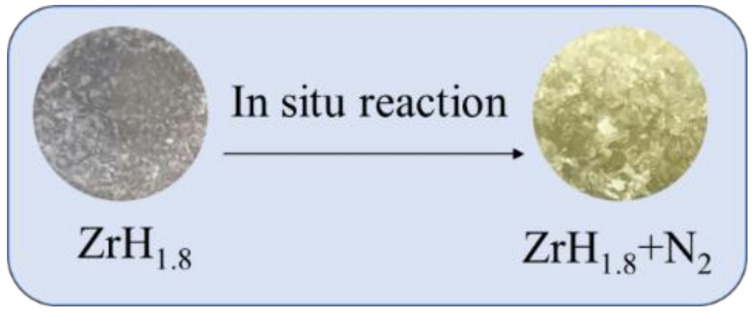
The appearance of the zirconium hydride sample before and after in situ reaction.

Figure 2 shows the appearance of the zirconium hydride sample before and after the in situ reaction. The surface of the original zirconium hydride sample was gray with a metallic luster, and became golden after the in situ reaction. Niyomsoan et al. [28] showed that the color of the zirconium nitride varies with the nitrogen partial pressure, and the typical color of zirconium nitride was golden. It was inferred that the films containing zirconium nitride were grown on the surface after the in situ reaction of nitrogen with zirconium hydride.

**Figure 3:**
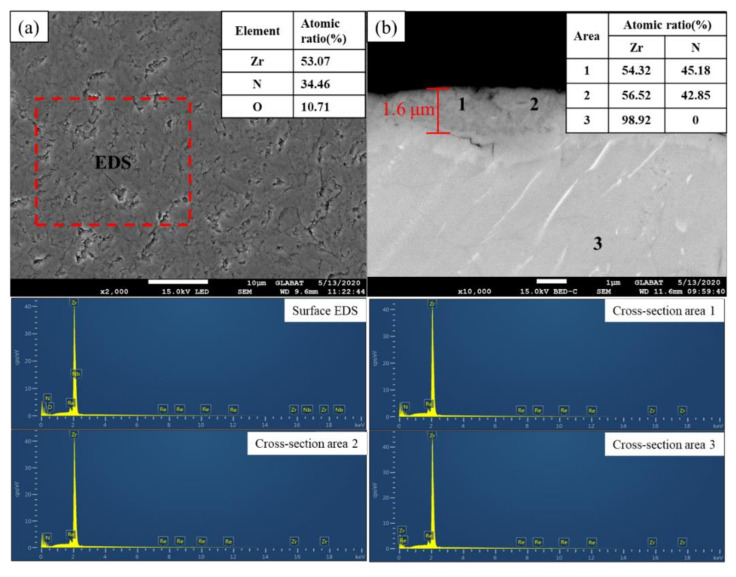
SEM images of the films: (**a**) surface morphology; (**b**) cross-section morphology.

Figure 3 shows the results of SEM and EDS analysis of the films on zirconium hydride after the in situ reaction. Figure 3a showed the surface of the sample was smooth, continuous, and dense. The EDS result showed that there were Zr, N, and O on the surface of the sample, which indicated that zirconium nitride and zirconium oxide films were possibly formed. Figure 3b showed cross-section morphology, in which it can be seen that the film was continuous and compact, tightly combined with the substrate, and the thickness was about 1.6 μm. N and Zr elements were detected in the cross-section regions 1 and 2 of the film with about 1:1 N/Zr atomic ratio, indicating that the film was possibly composed of ZrN, and only Zr element was detected in the matrix region 3, corresponding to the ZrHx composition of the matrix.

### 3.2. Phase Structure

**Figure 4:**
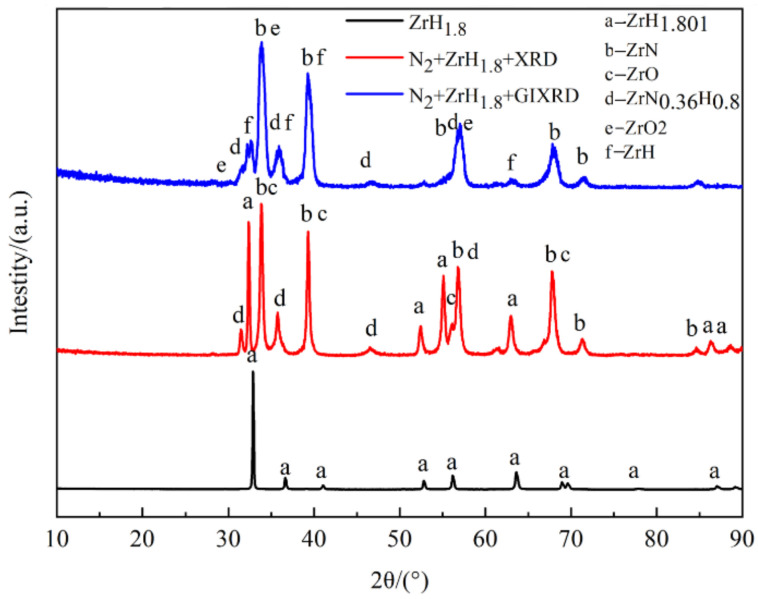
XRD spectrum of zirconium hydride before and after in situ reaction.

Figure 4 and Table 3 show the XRD patterns of the blank ZrH_1.8_, conventional XRD of the nitride film, and grazing incidence XRD (GIXRD) of nitride film. The phase of the blank ZrH_1.8_ was ZrH_1.801_; the phases of the films by conventional XRD analysis were ZrH_1.801_, ZrN, ZrO, and ZrN_0.36_H_0.8_; and the phases of the films by GIXRD analysis were ZrH, ZrN, ZrO_2_, and ZrN_0.36_H_0.8_. The existence of ZrH_1.801_ peak was caused by the X-ray penetration through the thin film. The existence of ZrN, ZrO_x_ (x = 1 or 2), and ZrN_0.36_H_0.8_ phases in the conventional XRD and GIXRD analysis indicated that a composite film was formed on the surface of the zirconium hydride by in situ reaction of nitrogen with zirconium hydride, where the O may be originated from the zirconium hydride matrix, impurities of the nitrogen or introduced during sealing. It can be seen from the figure that the main peak is ZrN. The detection of ZrN_0.36_H_0.8_ phase indicated that the nitrogen in the films may capture hydrogen penetrated from zirconium hydride matrix and form N-H bond, revealing the hydrogen barrier mechanism of nitride film.

### 3.3. Element Distribution

**Figure 5:**
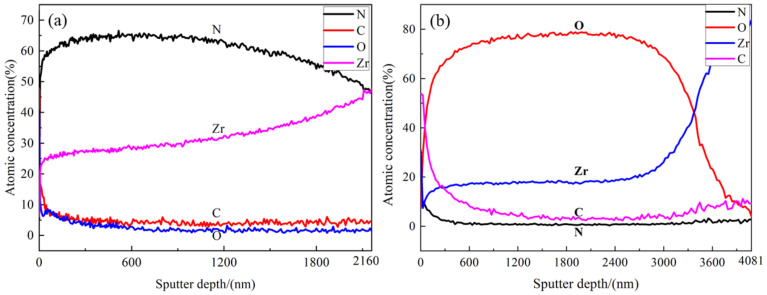
AES element distribution in the film with sputtering depth: (**a**) nitride film prepared in this paper; (**b**) oxide film prepared by Yan et al. [21].

Figure 5 displays the AES element distribution in the film with sputtering depth; Figure 5a represents the nitride film prepared in this paper; Figure 5b indicates the oxide film prepared by Yan et al. [21]. Figure 5a shows that the main elements of the nitride film were N and Zr, and the oxygen in the film might be derived from the impurities of the nitrogen and the affinity of oxygen with Zr, and the existence of C was caused by the contamination of the sample. With the increase of the sputtering depth, the content of Zr increased; while the content of N increased first but then decreased, with the highest N content of about 65% at a depth of 500 nm. The content of O decreased to zero when the sputtering depth was greater than 350 nm. Thus, it can be concluded that the diffusion depth of nitrogen was deeper than that of oxygen, which might be due to the fact that the in situ reaction was carried out in nitrogen atmosphere. The content of Zr and N were equal at the depth of 2160 nm, corresponding to the composition of ZrN that was detected by XRD analysis; and the elements composition in the film by AES analysis were coherent with the SEM and XRD results.

The composition of the film of this study was compared with that of the previously studied sample that was formed by the in situ reaction with urea at 600 °C, as shown in Figure 5b [21]. Both the films were composed of C, N, O, and Zr; however, N was the main interstitial element in the film of this study, and O was the main interstitial element in the film of the previous study, and the content of C in the film of this study is obviously lower than that of the previous study with urea as the reaction material. The reason for the composition difference between these two kinds of films was that the nitride film of this study was prepared in nitrogen atmosphere with residual content of oxygen at 800 °C, whereas the film of the previous study was prepared through the pyrolysis of urea at 600 °C which provided relatively more O and C and less N. In addition, the higher temperature provided a better diffusion condition for N in the film in this study.

### 3.4. Valence State

**Figure 6:**
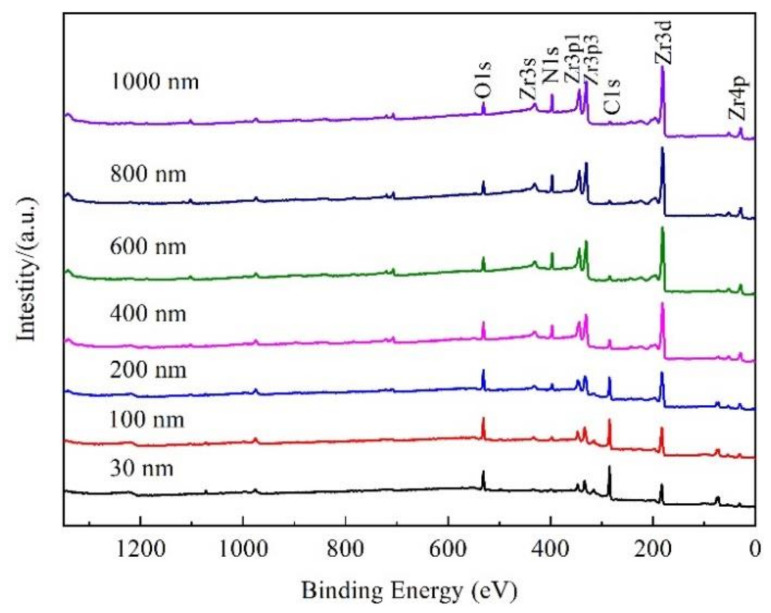
Full XPS spectrum of nitride film with sputtering depths.

XPS was used to analyze the elements content and their valence state in the film. XPS sputtering depth of 0–1000 nm was selected in the analysis. Figure 6 shows the full XPS spectrum of nitride film at different sputtering depth. The peak of C gradually disappeared with the increase of sputtering depth. The peaks of Zr and N intensified with the increase of the sputtering depth when the sputtering depth was less than 200 nm. However, the peaks of Zr and N showed no obvious change in the sputtering depth from 400 nm to 1000 nm, which was consistent with the results of the AES in Figure 5. In addition, the peak of O showed little change with sputtering depth from 30 nm to 1000 nm.

**Figure 7:**
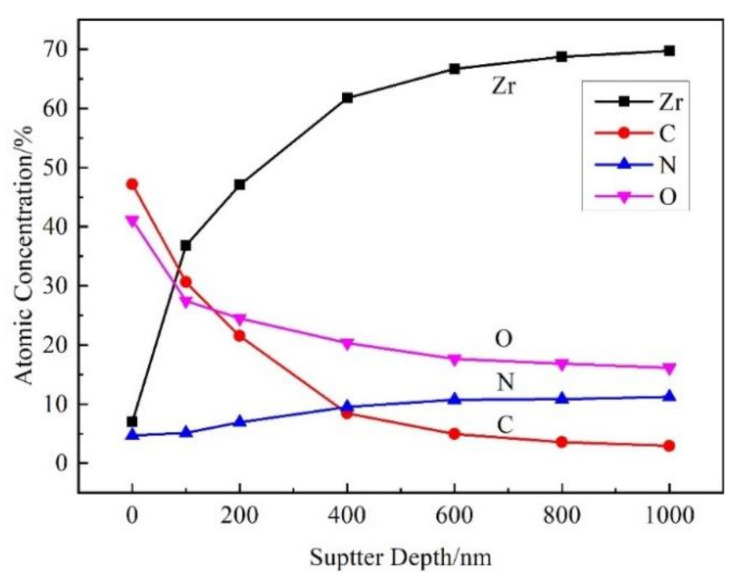
The element distribution of nitride film with different sputtering depths.

The element content at different depths of the film was calculated from the XPS peak area. Figure 7 shows the atomic concentration of Zr, C, N, and O in the film at different sputtering depths. With the increase of the sputtering depth, the content of Zr and N increased, while those of C and O decreased, which was consistent with the results of the AES result in Figure 5a. The content of C was zero when the sputtering depth exceeded 400 nm. The content of N was lower than that of O in XPS analysis in Figure 7, whereas the content of N was higher than that of O in AES analysis in Figure 5a, owing to the different analysis methods. Through literature [12,20,29] and consultation with XPS analysis specialist, the XPS was mainly used to determine the valence state of elements qualitatively, the content analysis was semi quantitative and less applied, and the quantitative analysis result of XPS was not as reliable as that of AES.

**Figure 8:**
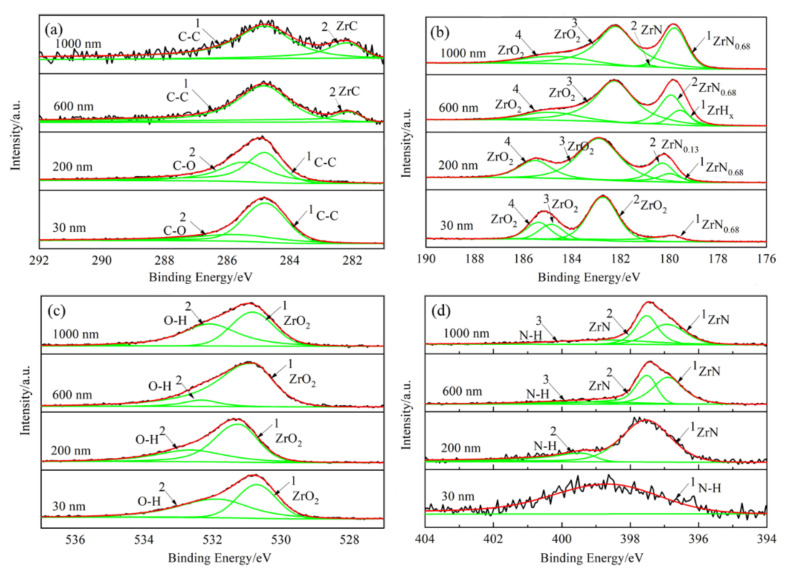
XPS narrow spectrum of C1s, Zr3d, O1s, and N1s at different sputtering depths. (**a**) C1s; (**b**) Zr3d; (**c**) O1s; (**d**) N1s.

The XPS narrow-spectrum analysis was measured at 30 nm, 200 nm, 600 nm, and 1000 nm, respectively, and the binding energy of C1s (284.8 eV) was used to calibrate the XPS peaks. Figure 8 showed the XPS narrow spectrum of C1s, Zr3d, O1s, and N1s at different sputtering depths, and the peak values were fitted in Table 4. C1s was all decomposed into two peaks at different depths, and the peak at 284.8 eV was C-C, and at 282.1 eV was assigned to ZrC, and the peak at 286 eV might refer to C-O [30]. There were four peaks at each depth after fitting the peak of Zr3d, implying that the films were zirconia and zirconium nitride, and the peaks of zirconium nitride increased with the increase of the sputtering depth, which was consistent with the AES results in Figure 5. There were two peaks at each depth after fitting the peak of O1s, the first peak corresponded to ZrO_2_ at different depths, and the second peak referred to O-H bond [21,31]. The peak of N1s was similarly consulted [32], and the results showed that nitrogen in the film was in the form of N-H and ZrN. The formation of O-H and N-H bonds in the film indicated that the oxygen and nitrogen captured the hydrogen from the zirconium hydride, and revealed the mechanism of hydrogen permeation. Compared with XPS, electron energy loss spectroscopy (EELS) can also be used for element content and valence analysis with higher resolution and accuracy; local chemical variations of H were recently measured by it [33,34], and will be used in the study of films prepared on zirconium hydride later.

### 3.5. Hydrogen Permeation Behavior

The hydrogen permeation resistance performance of nitride film was studied by three different methods including (1) PRF; (2) VTD; (3) HPR, and the results were compared with oxide film prepared by in situ reaction with CO_2_ [18].

#### 3.5.1. Permeation Reduction Factor

PRF measurement results of zirconium hydride samples with two different films (nitride and oxide films) are given in Table 5. The PRF values of the oxide and nitride films were 3.48 and 9.40 respectively, indicating that the hydrogen permeation resistance effect of nitride film was better than that of oxide film. The PRF value of zirconium hydride with nitride and oxide films was lower than stainless steel with films [9,10,11,12,13,14,15,16,17], which is because zirconium hydride is a brittle compound and surface defects are inevitable, and the quality of film on zirconium hydride must be much inferior than that on stainless steel.

#### 3.5.2. Vacuum Thermal Dehydrogenation

**Figure 9:**
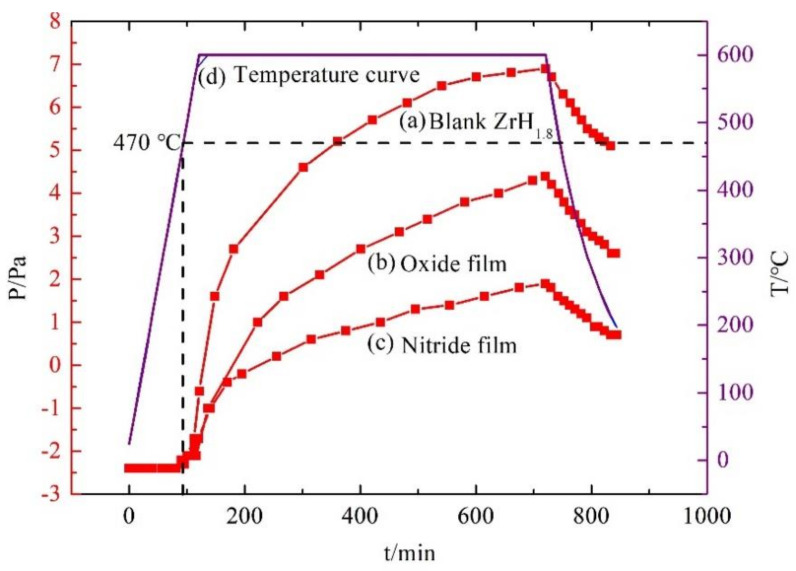
t-T-P curves of different films by vacuum thermal dehydrogenation: (a) Blank ZrH_1.8_; (b) Oxide film; (c) Nitride film; (d) Temperature curve.

The pressure changes with treatment time for bare ZrH_1.8_, ZrH_1.8_ with oxide film, and ZrH_1.8_ with nitride film are shown in Figure 9(a–c) respectively, and the temperature change with treatment time is shown in Figure 9(d) in the vacuum thermal dehydrogenation experiments. It can be seen that when the temperature was lower than 470 °C, the pressure of the three different samples did not change during heat treatment, indicating that the dehydrogenation temperature of zirconium hydride was higher than 470 °C. With the increase in temperature from 470 °C to 600 °C and during the 10 h holding period at 600 °C, the pressure of the three different samples increased continuously, indicating that gas emission occurred. The pressure value from high to low of the three kinds of samples showed an order of bare ZrH_1.8_ > ZrH_1.8_ with oxide film > ZrH_1.8_ with nitride film. Under vacuum condition, the pressure represented the amount of hydrogen loss, and the greater the pressure was, the more the hydrogen loss was. Therefore, the hydrogen permeation resistance effect of nitride film was better than that of oxide film, which was consistent with the PRF value results.

#### 3.5.3. Hydrogen Permeation Rate

**Figure 10:**
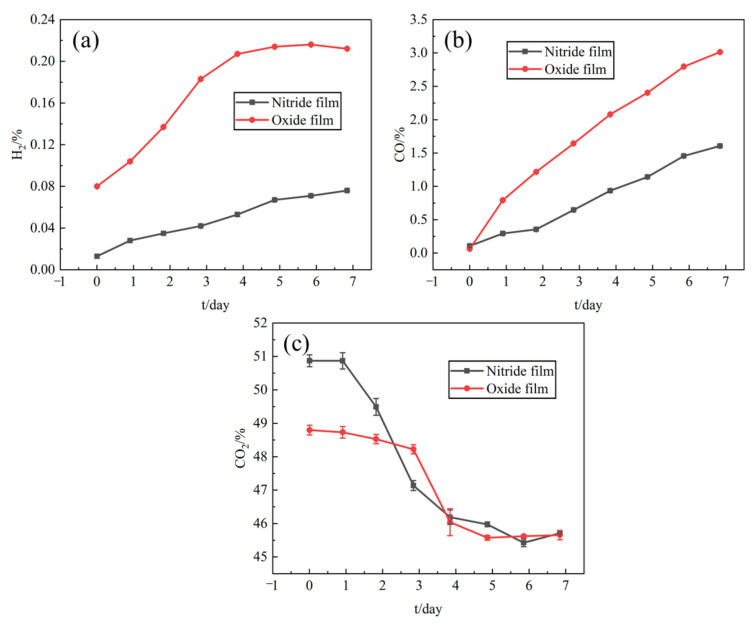
Variation of H_2_, CO, and CO_2_ content with time of different films. (**a**) H_2_; (**b**) CO; (**c**) CO_2_.

The zirconium hydride samples with nitride film and oxide film were tested in He + 50% CO_2_ atmosphere at 600 °C, and the content changes of H_2_, CO, and CO_2_ with treatment time are shown in Figure 10. It could be seen that the contents of H_2_, CO, and CO_2_ for zirconium hydride samples with two different films had the same variation trend with the increase of treatment time. The CO_2_ content kept decreasing with treatment time; the contents of CO and H_2_ increased with the treatment time, but these contents in the sample with the oxide film were obviously higher than that of the nitride film, indicating that the nitride film had better hydrogen permeation resistance effect.

Based on the measured contents of CO and H_2_ calculated by the model developed by Qi et al. [22] and Bai et al. [27], the hydrogen permeation rate Va (kg·m^−2^·s^−1^) was calculated and is listed in Table 6.

**Figure 11:**
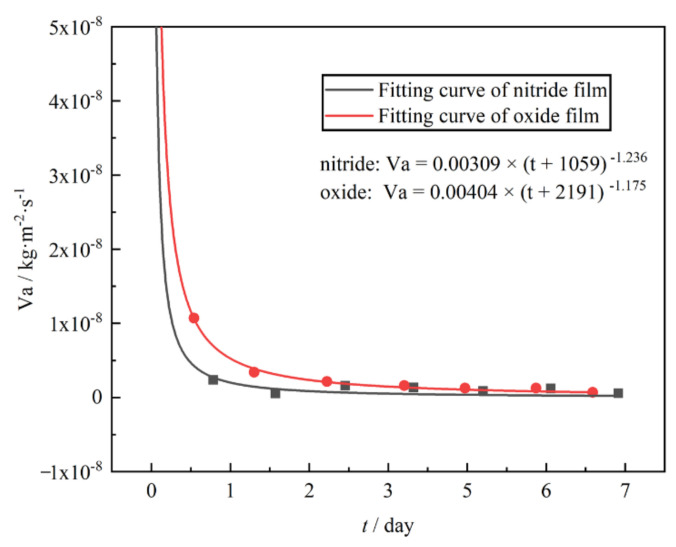
Fitting diagram of hydrogen permeation rate of different films.

Figure 11 shows the fitting results of hydrogen permeation rates of the two films, and the results demonstrate that the relationship between hydrogen permeation rate Va (kg·m^−2^·s^−1^) and time t (s) for nitride film and oxide film conformed to Equations (4) and (5), respectively:

(4) $$V_{a}=0.00309\times {\left(t+1059\right)}^{-1.236}$$

(5) $$V_{a}=0.00404\times {\left(t+2191\right)}^{-1.175}$$

According to Equations (4) and (5), the hydrogen loss over a long period of time was predicted and listed in Table 7. It can be found that the hydrogen loss value of nitride film was 3.179%, 3.228%, and 3.254%, while that of oxide film was 7.069%, 7.259%, and 7.360% for 1, 2, and 3 years, respectively. The predicted hydrogen loss values of oxide film were higher than that of nitride film, indicating that the nitride film exhibited better hydrogen permeation resistance effect than the oxide film. This result supported the results of PRF and VTD methods.

## 4. Conclusions

In this work, a composite nitride film was prepared on zirconium hydride by the in situ reaction method in a nitrogen atmosphere.

The film was golden color, smooth, continuous, and dense, with a thickness of about 1.6 μm. XRD analysis showed that the main phase of the film was ZrN, with coexistence of ZrO_2_, ZrO, and ZrN_0.36_H_0.8_. The element distribution of nitride film was compared with that of previously studied by in situ reaction with urea at 600 °C by AES analysis. Two types of films were composed of elements C, N, O, and Zr; the content of N in the film of this study was the main interstitial element, but O was the main interstitial element in the film of the previous study, and the content of C in the film of this study was obviously lower than that of the previously studied with urea as the reaction material. XPS analysis revealed that there were Zr-C, Zr-O, Zr-N, O-H, N-H bonds in the film, which further verified the results of AES analysis. Based on these characterization results, the hydrogen barrier mechanism could be attributed to the existence of the ZrN_0.36_H_0.8_ phase and the bonds of O-H and N-H, indicating that the nitrogen and oxygen in the film could capture the hydrogen from zirconium hydride matrix.

Finally, the hydrogen permeation resistance performance of the nitride film was compared with that of oxide film by PRF, VTD, and HPR methods, and the results showed that the hydrogen permeation barrier effects of nitride film was better than that of oxide film. This study investigated the preparation and hydrogen permeation behavior of zirconium nitride film on zirconium hydride and revealed the hydrogen barrier mechanism of nitride films.

## Figures and Tables

**Table 1:** Contents of impurity elements in zirconium hydride.

Element	Fe	Hf	Nb	O	N
Content/ωt%	0.003	0.0052	0.96	0.0056	0.0043

**Table 2:** Samples investigated for hydrogen permeation behavior.

Sample	Film	Initial Thicknesses
ZrH_1.8_ with nitride film	ZrN	1.6 μm
ZrH_1.8_ with oxide film	ZrO_2_	2 μm

**Table 3:** XRD results before and after in-situ reaction of zirconium hydride in nitrogen atmosphere.

Sample	Phase			
Blank ZrH_1.8_	ZrH_1.801_			
XRD of film	ZrH_1.801_	ZrN	ZrN_0.36_H_0.8_	ZrO
GIXRD of film	ZrH	ZrN	ZrN_0.36_H_0.8_	ZrO_2_

**Table 4:** Binding energy values of XPS narrow spectrum of the nitride film.

Elements	h/nm	Peak1/eV	Peak2/eV	Peak3/eV	Peak4/eV
C1s	30	284.78	285.67	-	-
	200	284.80	285.42	-	-
	600	284.82	282.12	-	-
	1000	284.82	282.17	-	-
Zr3d	30	179.84	182.70	184.85	185.36
	200	179.94	180.27	182.89	185.52
	600	179.55	179.92	182.24	184.84
	1000	179.77	181.14	182.23	184.60
O1s	30	530.71	531.83	-	-
	200	531.24	532.64	-	-
	600	530.82	532.32	-	-
	1000	530.84	532.09	534.66	-
N1s	30	398.67	-	-	-
	200	397.53	399.42	-	-
	600	396.90	397.51	399.27	-
	1000	396.92	397.51	398.82	-

**Table 5:** PRF values of zirconium hydride samples with different films.

Samples	m_1_/g	m_2_/g	Δm/g	Δm/S g·m^−2^	PRF	Average PRF	Standard Deviation
ZrH_1.8_	5.833	5.825	0.008	3.7230	-	-	-
	20.0762	20.0625	0.0137				
	20.0358	20.0195	0.0163				
Oxide film	5.4483	5.4447	0.0036	1.1983	3.1069	3.4811	0.4900
	19.7596	19.7555	0.0041	1.1770	3.1631		
	19.6229	19.6198	0.0031	0.8921	4.1733		
Nitride film	5.3744	5.3735	0.0009	0.2962	12.5692	9.3970	2.3157
	19.1102	19.1087	0.0015	0.4372	8.5156		
	19.1605	19.1587	0.0018	0.5239	7.1063		

**Table 6:** Hydrogen permeation rate of different films.

Nitride Film			Oxide Film		
t/day	Va/kg·m^−2^·s^−1^	σ_1_	t/day	Va/kg·m^−2^·s^−1^	σ_2_
0.003	4.309 × 10^−7^	1.67867 × 10^−8^	0.003	4.220 × 10^−7^	2.78229 × 10^−9^
0.907	2.364 × 10^−9^	2.82329 × 10^−11^	0.625	1.072 × 10^−8^	2.09711 × 10^−10^
1.820	5.344 × 10^−10^	3.29286 × 10^−11^	1.509	3.424 × 10^−9^	1.38303 × 10^−10^
2.841	1.596 × 10^−9^	2.22545 × 10^−11^	2.575	2.167 × 10^−9^	6.02349 × 10^−11^
3.843	1.354 × 10^−9^	7.21309 × 10^−11^	3.707	1.608 × 10^−9^	1.38992 × 10^−11^
4.862	8.778 × 10^−10^	3.29768 × 10^−11^	4.598	1.265 × 10^−9^	4.48827 × 10^−11^
5.855	1.201 × 10^−9^	9.16133 × 10^−12^	5.640	1.260 × 10^−9^	1.38900 × 10^−11^
6.843	5.707 × 10^−10^	3.71908 × 10^−11^	6.470	6.866 × 10^−10^	4.30914 × 10^−11^

**Table 7:** Fitting formula of different films and prediction of hydrogen loss in 1–3 years.

Film	Va/kg·m^−2^·s^−1^	R^2^	1 Year	2 Year	3 Year
Nitride	$V_{a}=0.00309\times {\left(t+1059\right)}^{-1.236}$	0.99998	3.179%	3.228%	3.253%
Oxide	$V_{a}=0.00404\times {\left(t+2191\right)}^{-1.175}$	1	7.069%	7.259%	7.360%

## Data Availability

The data presented in this study are available upon request from the corresponding author.
